# 2222. 3-Day Ceftriaxone vs. Longer Durations of Therapy For Inpatient Treatment of Uncomplicated UTI 

**DOI:** 10.1093/ofid/ofac492.1841

**Published:** 2022-12-15

**Authors:** Balsam Elajouz, Lisa E Dumkow, Kali VanLangen, Lacy Worden, Andrew Jameson

**Affiliations:** Trinity Health St. Mary's Grand Rapids, Comstock Paek, Michigan; Trinity Health Saint Mary's Grand Rapids, Grand Rapids, MI; Trinity Health St. Mary's Grand Rapids, Comstock Paek, Michigan; Mercy Health Saint Mary's, Lowell, Michigan; Trinity Health Saint Mary's Grand Rapids, Grand Rapids, MI

## Abstract

**Background:**

In the outpatient setting, IDSA guidelines recommend a 3-day course of highly bioavailable oral antibiotics (abx) for treatment of uncomplicated urinary tract infections(uUTI); however, hospitalized patients often receive IV abx and longer durations. Ceftriaxone (CRO) is well tolerated and has a favorable spectrum of activity making it an ideal antibiotic for inpatient uUTI treatment. The purpose of this study was to compare a short, 3-day course of ceftriaxone with longer durations of therapy for inpatients with uUTI.

**Methods:**

This retrospective cohort study included adult inpatients receiving abx for symptomatic uUTI with a positive urine culture between July 1, 2015 and June 30, 2021. The primary objective was to compare clinical cure between patients treated with 3-days of CRO (CRO 3-day) vs longer durations of abx therapy (Longer DOT). Clinical cure was defined as resolution of uUTI symptoms at completion of abx and no new documented symptoms within 24 hours following completion. Secondary outcomes included comparing hospital length of stay (LOS), 30-day return visit due to UTI, and development of Clostridiodes difficile within 30 days of abx treatment. Patients empirically treated with an anti-pseudomonal abx, microbiological resistance to CRO, co-infection, or complicated UTI were excluded.

**Results:**

A total of 100 patients were included in the study (CRO 3-days, n=51; Longer DOT, n=49). Baseline characteristics were similar between groups. There was no difference in the primary endpoint between groups as all patients in the sample population achieved clinical cure (p=1.0). Additionally, no differences in median hospital LOS (5 [4-7] vs 4 [3-6.5] days, p=0.372), 30-day return visit due to UTI (13.7% vs 6.1%, p=0.319), or development of C. difficile within 30 days of treatment (2% vs 6.1%, p=0.357) were observed between the CRO 3-days vs Longer DOT groups, respectively.

**Conclusion:**

There were no differences in efficacy or safety endpoints between short-course CRO and longer durations of therapy. Short-course IV CRO is likely an effective treatment strategy for inpatient treatment of uUTI and may limit prolonged antibiotic durations.

**Disclosures:**

**All Authors**: No reported disclosures.

